# Relationship of primary immune thrombocytopenic purpura and atopia among children: a case control study

**DOI:** 10.1038/s41598-020-68647-2

**Published:** 2020-07-16

**Authors:** Gholamreza Bahoush, Amirbahador Poorasgari, Marzieh Nojomi

**Affiliations:** 1 Department of Pediatrics, Faculty of Medicine, Ali-Asghar Children Hospital, Iran University of Medical Sciences, Tehran, Islamic Republic of Iran; 2 Preventive Medicine and Public Health Research Center, Psychosocial Health Research Institute, Department of Community and Family Medicine, School of Medicine, Iran University of Medical Sciences, Tehran, Iran

**Keywords:** Biological techniques, Chemical biology, Immunology, Physiology, Diseases, Health care, Medical research

## Abstract

Atopic dermatitis (AD) is a chronic disease affecting 10–30% of children and 2–10% of adults worldwide. It is manifested by the pruritus eczema lesions on the skin. Immune thrombocytopenic purpura (ITP) is the most common cause of acute onset of thrombocytopenia in childhood. The aim of this study was association of primary immune thrombocytopenic purpura and atopia among children. This case control study was performed on patients with acute and chronic ITP who were confirmed by a hematologist. The control group was also selected from the siblings of the patients who were healthy and almost matched by age and sex with the patient group. Data were entered into a questionnaire under the SPSS-20 program, and demographic data were analyzed descriptively. In the present study, 120 patients were enrolled, 60 of whom were in the patient group and 60 in the control group. Mean age was 95 and 98 months for patients and control. This study showed a significant association of ITP with allergic rhinitis (P = 0.02), atopic dermatitis (P = 0.004), itching (P = 0.042), and dry skin (P = 0.015). However, no significant relationship was found between ITP and asthma (P-value = 0.18). This study does not reveal the causality between atopy and ITP but clearly shows the association between atopy and ITP disease, so the prevalence of atopy in ITP patients is higher than the normal population. According to the results of this study, it is necessary to investigate the cause of atopy and ITP and to find other immunological and possibly genetic commonalities.

Atopic dermatitis is the most common chronic inflammatory skin disease, affecting about 10–30% of children and 2–10% of adults, thus making it a major global health problem. Onset of atopic dermatitis is typically present in infancy, which is characterized by recurrent periods of rash, dryness, and itch^1^. Symptoms occur in 45% of patients within the first 6 months of life, and face and neck are involved in more than 90% of them. Another point to note is that 60% of infants and children with this disease recover up to 12 years of age and rarely persist until adulthood^2,3^.

Atopic dermatitis is a complex genetic disorder that is often associated with other atopic disorders, such as allergic conjunctivitis, asthma, allergic rhinitis, or food allergies. These genetic disorders are prominent in infants and young children as atopic dermatitis, in older children asthma and in adolescents as inhaled allergies^4–6^. The term atopic refers to the genetic tendency for allergenic disorders with eosinophilia in all patients, and elevated serum IgE levels (between 70 and 80%) in most patients^7,8^. The manifestations of the disease and the location of the lesions vary with age and can be manifested by skin, respiratory and food allergies and mucosal involvement^9,10^. The main treatment for the disease is topical corticosteroids, which are prescribed based on the severity of the lesions with low, moderate or severe potency^11^. Atopic disorders such as allergic conjunctivitis, asthma, allergic rhinitis can occur in other diseases such as Immune thrombocytopenic purpura (ITP). ITP is one of the most common causes of thrombocytopenia and is a syndrome that causes platelet dysfunction^12^. Antiplatelet antibodies secreted by autoreactive B lymphocytes destroy platelets in the reticuloendothelial system, especially the spleen by binding to platelet antigens, which is described as the major immunological defect in ITP^13^.

The peak of the disease is between 1 and 2 years. Although it is limited in age from infancy to adulthood, boys and girls are treated equally in childhood. ITP is clinically common with petechia, purpura, and mucosal bleeding that usually results from an upper respiratory tract infection^14^. It manifests itself in the majority of affected children with acute self-limitation up to 12 months, with or without treatment, and eventually the platelet count returns to normal^15,16^. There are several risk factors for chronicity of the disease, one of which is initial lymphocyte count^17^. Other studies have provided evidence of a chronic humoral deficiency in chronic cases^18^. A growing body of evidence indicates that there is a link between autoimmune diseases and allergic diseases. However, few studies have evaluated the association between allergic diseases and ITP. In a study of children with AD, the incidence of ITP was higher in people with AD than in non-AD patients. It is also more likely to develop ITP in people with AD within the first 3 years after diagnosis of AD. Other autoimmune disorders in individuals with ITP and AD are more likely than those with ITP who do not have AD^19^. Another study also shows that ITP increases the risk of allergic diseases such as AD in children^20^.

Given the immunological abnormalities in the pathophysiology of both diseases, the association of both diseases with allergic diseases, and the presence of genetic risk factors in both diseases, we aimed to investigate the relationship between these two disorders in terms of epidemiology and clinical manifestations of atopy.

## Methods

### Study design

This case control study was performed on patients with acute and chronic ITP (treated or untreated patients) at Hospital, who were confirmed by a hematologist to rule out other causes. In order to rule out of Wiscott–Aldrich syndrome, only patients who had higher mean platelet volume (MPV) than normal at the time of diagnosis were enrolled. In this study, age and sex distribution are evenly matched. The control group was even selected as possible from the siblings of the patients who were healthy and almost similar in age and sex to the patient group. Questionnaires were prepared for both types of atopy based on demographic, acute or chronic disease and standard criteria in both groups and then completed by a pediatric immunologist. The sampling method was non-probable and based on a questionnaire. Considering the prevalence of 5–20% of atopy in the literature, the sample size for each of the two case and control groups was estimated at 60 (power = 80%, alpha = 0.05) to make a difference of at least 20% between the two groups.

### Data analysis

Data were entered into a questionnaire under the SPSS-20 program, demographic data were analyzed descriptively and statistical comparison between the two groups was done by Chi-square test. P-value < 0.05 was defined as significant.

### Ethical statement

All participants provided written informed consent. The study was conducted in accord-ance with the Declaration of Helsinki.All procedures were approved by the Human Research Committee of Iran University of Medical Sciences.

## Results

A total of 120 patients were enrolled in this study, 60 in the patient group and 60 in the control group. Mean age of patients in patients and control groups was 95 and 98 months. The study subjects consisted of 32 boys and 28 girls (Table 1).

**Table 1:** Evaluation of allergic rhinitis and ITP.

Allergic rhinitis	Group			
	Patients		Control	
	Frequency	Percent	Frequency	Percent
Yes	23	38.30	12	20
No	37	61.70	48	80
Total	60	100	60	100

Regarding the association between asthma and ITP in the patient and control groups, the results showed that, only 4 cases (6.3%) had asthma in the patient group and 56 cases (93.7%) did not have asthma. Furthermore, 59 (98.3%) showed asthma in the control group and only 1 (1.7%) had asthma. Statistically, it can be concluded that there is no relationship between asthma and ITP (P = 0.18). The relationship between allergic conjunctivitis and ITP was indicated in Table 2 and the results showed that the frequency of allergic conjunctivitis among patients with ITP was ten times higher than that of the control group. Conjunctival disorders in patients with ITP is more likely than control group (P = 0.05) (Table 2).

**Table 2:** Relationship between allergic conjunctivitis and ITP.

Allergic conjunctivitis	Group			
	Patients		Control	
	Frequency	Percent	Frequency	Percent
Yes	6	10	1	1.70
No	54	90	59	98.30
Total	60	100	60	100

The results showed that 14 patients (23.3%) were affected and 46 cases (76.7%) did not affected by atopic dermatitis. In addition, in the control group, 57 cases (95%) had no atopic dermatitis and only 3 cases (5%) developed atopic dermatitis. Results show that atopic dermatitis is more common in the ITP group as compared to control group (P = 0.004).

The association between ITP and the itch is shown in Table 3, indicating a low incidence of itching in infants with ITP. The absence of even one case in the control group indicates that the difference was statistically significant among both group (P = 0.04), indicating that infants with ITP exhibited more pruritus than controls (Table 3).

**Table 3:** Neonatal itch and ITP.

Neonatal itch	Group			
	Patients		Control	
	Frequency	Percent	Frequency	Percent
Yes	4	6.70	0	0
No	56	93.3	60	100
Total	60	100	60	100

In the patient group, 10 cases (16.7%) had dry skin and the other 50 neonates (83.3%) were not affected. In the control group, 58 (96.7%) had no dry skin and only 2 (3.3%) experienced the complication. Therefore, there was a significant relationship between the prevalence of skin dryness in infants and ITP (P = 0.015), indicating that dry skin in infants with ITP was more common than the control group.

In addition, Atopic dermatitis, infant dry skin and pruritus were more frequent among acute ITP enrolled patients versus control group; these differences were statistically significant. On the other hand, This statistically significant difference was found only for orbital darkening among chronic ITP enrolled patients versus control group (Table 4).

**Table 4:** Frequency of important Atopia stigmata among acute and chronic ITP enrolled patients versus control group.

Clients	Allergic rhinitis		P value
	Yes	No	
Acute ITP	11 (36.7%)	19 (63.3%)	0.104
Chronic ITP	12 (40%)	18 (60%)	0.058
Control	13 (21.7%)	47 (78.3%)	
	Infant dry skin		
Acute ITP	8 (26.7%)	22 (73.3%)	0.002
Chronic ITP	2 (6.7%)	28 (93.3%)	0.407
Control	2 (3.3%)	58 (96.7%)	
	Infant eczema		
Acute ITP	5 (16.7%)	25 (83.3%)	0.078
Chronic ITP	4 (13.3%)	26 (86.7%)	0.164
Control	3 (5%)	57 (95%)	
	Atopic dermatitis		
Acute ITP	9 (30%)	21 (70%)	0.005
Chronic ITP	5 (16.7%)	25 (83.3%)	0.133
Control	4 (6.7%)	56 (93.3%)	
	Infant pruritis		
Acute ITP	3 (10%)	27 (90%)	0.035
Chronic ITP	1 (3.3%)	29 (96.7%)	0.333
Control	0 (0%)	60 (100%)	
	Orbital darkening		
Acute ITP	2 (6.7%)	28 (93.3%)	0.257
Chronic ITP	4 (13.3%)	26 (86.7%)	0.041
Control	1 (1.7%)	59 (93.3%)	

## Discussion

As expected, there was a strong significant association between ITP as an autoimmune disorder and atopic dermatitis as an immunological disorder. The results of our study, despite the low number of cases compared to other studies, show a strong significant relationship between these two immunological disorders. The severity of this relationship varies according to the type of atopic manifestation and the highest rate was associated with atopic dermatitis and the lowest was related to allergic conjunctivitis.

However, no significant relationship was found between ITP and asthma (P-value = 0.18), which was consistent with the findings of Chiang et al. (2015), in which 1,203 children with ITP younger than 18 y of age were included between 1998 and 2008, as well as 4,812 individuals as control group.

The aforementioned study showed that the history of allergic diseases was much more common in children with ITP than in the control group. This comparison was performed for various allergic diseases such as allergic rhinitis (17.2 vs. 8.96%), asthma (8.65 vs. 5.24%), allergic conjunctivitis (8.98 vs. 5.36%), urticaria (4.16 vs. 2.41%), and atopic dermatitis (6.98 vs. 3.66%)^13^.

A cohort analysis study by Wei et al., in 2016, to investigate children with AD in comparison with non-AD controls revealed that from 2000 to 2007, 120,704 children with newly diagnosed AD and 241,408 children as non-AD controls were investigated for the risk of ITP.Finally, they showed that the incidence of ITP in AD patients was 1.72 times higher than in the control group, which was 2.4 times higher in children older than 2 years and gradually decreased with increasing age^21^. These findings were consistent with our findings.

Paola Giordano et al., in a cohort retrospective analytic study on a large number of children with chronic ITP for the prevalence of positivity of antithyroid antibodies demonstrated that the significantly higher prevalence of positivity of antithyroid antibodies (antiTPO and anti-TG) in pediatric patients with chronic ITP in comparison with the pediatric population^22^.

In another case–control study by Giordano et al. Of three patients with relapsed ITP after splenectomy was confirmed in vitro hyperactive B cells and high numbers of antibody-producing cells after splenectomy^23^.

Surprisingly and may be contrary to expectation, in our study, we found a more significant correlation between acute ITP and Ethiopia versus chronic ITP. It may change in a study with a larger sample.

## Conclusion

The findings revealed a significant relationship between types of atopy (except for asthma) and ITP. This is one of the few studies on the relationship between atopy and ITP that can be very important in future studies. Other benefits of this study include the study of a atopic type such as atopic dermatitis, allergic rhinitis, allergic conjunctivitis, pruritus and neonatal dry skin, which is very comprehensive. The findings suggest a study of the types of possible genetic disorders and immune systems involved in the two diseases, which are identical, and that the results of these studies may pave the way for new therapies.
